# Analysis of the Potential Relationship between Aging and Pulmonary Fibrosis Based on Transcriptome

**DOI:** 10.3390/life12121961

**Published:** 2022-11-23

**Authors:** San Fu, Xiaoyan Tang, Yiming Xu, Xianrui Song, Xiuhui Qian, Yingying Hu, Mian Zhang

**Affiliations:** 1 School of Traditional Chinese Pharmacy, China Pharmaceutical University, Nanjing 211198, China; 2 School of Basic Medicine and Clinical Pharmacy, China Pharmaceutical University, Nanjing 211198, China

**Keywords:** pulmonary fibrosis, aging, senescence, transcriptome, bioinformatics, chemokines

## Abstract

Idiopathic pulmonary fibrosis (IPF) is an age-related interstitial lung disease with a high incidence in the elderly. Although many reports have shown that senescence can initiate pulmonary fibrosis, the relationship between aging and pulmonary fibrosis has not been explained systematically. In our study, young and old rats were intratracheally instilled with bleomycin (1 mg/kg), and the basic pathological indexes were determined using a commercial kit, hematoxylin, and eosin (H&E) and Masson’s Trichrome staining, immunohistochemistry, immunohistofluorescence, and q-PCR. Then, the lung tissues of rats were sequenced by next-generation sequencing for transcriptome analysis. Bioinformatics was performed to analyze the possible differences in the mechanism of pulmonary fibrosis between aged and young rats. Finally, the related cytokines were determined by q-PCR and ELISA. The results indicate that pulmonary fibrosis in old rats is more serious than that in young rats under the same conditions. Additionally, transcriptomic and bioinformatics analysis with experimental validation indicate that the differences in pulmonary fibrosis between old and young rats are mainly related to the differential expression of cytokines, extracellular matrix (ECM), and other important signaling pathways. In conclusion, aging mainly affects pulmonary fibrosis through the ECM–receptor interaction, immune response, and chemokines.

## 1. Introduction

Idiopathic pulmonary fibrosis (IPF) is a chronic, progressive, interstitial lung disease that is epidemiologically investigated and clinically observed in the middle-aged and elderly population [1,2,3]. In addition, animal models of pulmonary fibrosis show that symptoms in young mice subside with age, while those in older mice do not subside with age [4]. The development of pulmonary fibrosis is closely related to cellular senescence, which is also an important factor in causing pulmonary fibrosis [5,6]. Existing studies usually investigate individual genes or proteins related to aging or fibrosis and are not systematic or comprehensive enough to explain the link between the two mechanisms of aging and fibrosis. Systems biology is a good approach for connecting the complex interactions of differently sourced biological information, such as genes, proteins, and metabolites at various levels of biological samples, which can define the elements of the system and allow the systematic analysis of mechanisms from a holistic viewpoint [7,8].

Second-generation sequencing, also known as next-generation sequencing (NGS), can cover the whole genome and transcriptome because of its high content screening and high sensitivity and has been used in increasing numbers of genomic and transcriptomic studies [9,10]. In the present study, young and old rats with pulmonary fibrosis were researched using transcriptomics and bioinformatics for the analysis of the differences in the mechanism of pulmonary fibrosis between old and young rats.

## 2. Materials and Methods

### 2.1. Animal Treatments

Young SD rats (6–8 weeks old, 180–220 g) and old SD rats (13 months old, 360–700 g), all male, were purchased from Shanghai Sipur-Bikai Experimental Animal Co., Ltd., Shanghai, China (SCXK(Hu) 2018-0006). All rats were housed in a temperature- and humidity-controlled environment (25 ± 1 °C, 55 ± 5%) under a 12 h light/dark cycle with free access to food and water and were allowed to acclimatize for a week before the experiment. Young and old rats were divided into sham and pulmonary fibrosis (PF) groups. On day 0, the rats of the PF group were intratracheally injected with bleomycin (Yuanye Bio-Technology, Shanghai, China) (1 mg/kg), and the sham group was injected with the same volume of 0.9% normal saline. Finally, all rats were divided into 4 groups: a sham group of young rats (y-Sham), a PF group of young rats (y-PF), a sham group of old rats (o-Sham), and a PF group of old rats (o-PF). The body weights of all rats were recorded every day. On day 14, the rats were killed under anesthesia. The animal welfare and experimental procedures complied with the Guidelines for the Care and Use of Experimental Animals and the related ethical regulations of China Pharmaceutical University. The experiment protocols were reviewed and approved by the Institutional Ethical Committee of China Pharmaceutical University (permit No. SCXK (Hu) 2018-0006).

### 2.2. Histopathological, Immunohistochemical, and Immunohistofluorescent Examination

Lung tissues were fixed in 10% formalin for 48 h, dehydrated, then embedded in paraffin and cut into 4–5 μm-thick sections. For the histopathological assay, the sections were stained with hematoxylin and eosin (H&E) or Masson’s Trichrome, then examined and evaluated by professionals under light microscopy. The H&E scores were assessed according to the following criteria: (a) the thickening of the alveolar septum, (b) pulmonary congestion, (c) the infiltration of inflammatory cells in the lung trachea and perivascular system, and (d) the degeneration of bronchial epithelial cells. The scores for each of the above four items ranged from 0 (normal) to 5 (extremely severe injury) and were obtained by calculating the sum of the four scores. The area of collagen fibers (in blue) and total tissue area (excluding the blank area) in the upper, lower, left, right, and middle fields of each Masson-stained section were selected and calculated using ImageJ 1.53o software. Collagen deposition was expressed by CVF (collagen volume fraction): CVF (%) = collagen area/total tissue area × 100%.

For immunohistochemical (IHC) and immunohistofluorescent (IHF) assays, the sections were blocked with 3% H_2_O_2_ for 15 min and 5% bovine serum albumin for 10 min and then incubated with α-SMA, Collagen I, and CCR2 antibodies overnight at 4 °C. Next, the sections were incubated with HRP-conjugated secondary antibody or Alexafluor488 and Cy3-conjugated secondary antibody at 37 °C for 30 min, developed with (IHC) or without (IHF) DAB solution, and visualized under an Olympus microscope.

### 2.3. Hydroxyproline Determination and ELISA Assay

Hydroxyproline (HYP), a product of collagen hydrolysis, is one of the markers of pulmonary fibrosis. In our present study, HYP was determined by the alkaline hydrolysis method. HYP level in lung tissue was determined by a commercial HYP assay kit (Nanjing Jiancheng Bioengineering, Nanjing, China) using 100 mg of lung tissue according to the manufacturer’s instructions.

The cytokines in lung tissues were determined by ELISA assays using commercial ELISA kits (Lianke Biotech Co., Ltd., Hangzhou, China) according to the manufacturer’s instructions. Briefly, lung tissue was homogenized with PBS and centrifuged (12,000 g/min) for 10 min to obtain the supernatant and then incubated with antibodies in a microtiter plate. After incubation with HRP-conjugated streptavidin, the samples developed color in the presence of TMB and were terminated by stop solution. The OD values were detected at a wavelength of 450 nm.

### 2.4. Real-Time qPCR Assay 

The total RNA of lung tissues was extracted using Trizol (Vazyme, Nanjing, China) and reverse transcribed into the first strand of cDNA using HiScript Q RT SuperMix for qPCR (Vazyme, Nanjing, China). Real-time PCR amplification was performed to analyze the gene expression profiles in a BIO-RAD CFX connect q-PCR system (Bio-Rad, Hercules, CA, USA) with AceQ qPCR SYBR Green Master Mix (Vazyme, Nanjing, China). The reaction conditions of PCR amplification were as follows: initial denaturation at 95 °C for 5 min, 40 cycles of denaturation at 95 °C for 10 s, and denaturation at 60 °C for 30 s. The primers were purchased from Tsingke Biotechnology Co., Ltd. (Beijing, China), and the sequences are listed in Table S1.

### 2.5. RNA Extraction Library Construction and Sequencing

The total RNA of lung tissues was extracted using Trizol (Vazyme, Nanjing, China) and qualitatively controlled by NanoDrop ND-1000 microquantitative instrument. Then, the integrity of RNA was detected using an Agilent Bioanalyzer 2100 biological analyzer. The eukaryotic mRNA with PolyA was specifically enriched from the qualified total RNA by two rounds of purification using Dynabeads Oligo (dT) (Thermo Fisher, San Jose, CA, USA). The enriched eukaryotic mRNA was fragmented into short fragments at 94 °C for 5 min using Magnesium RNA Fragmentation Module (NEB, cat. e6150, Ipswich, MA, USA) and then used as a template for reverse transcription into the first strand of cDNA by SuperScript™ II Reverse Transcriptase (Invitrogen, cat.1896649, Waltham, MA, USA). Then, the U-labeled second-stranded DNA was synthesized using E. coli DNA polymerase I (NEB, cat.m0209, USA), RNase H (NEB, cat.m0297, USA), and dUTP solution (Thermo Fisher, cat. R0133, USA). An A-base was then added to the blunt ends of each strand, preparing them for ligation to the indexed adapters, and size selection was performed with AMPureXP beads. Next, the U-labeled second-stranded DNA was treated with UDG enzyme (NEB, cat.m0280, USA), and PCR amplification was performed to obtain a library with a fragment size of 300 ± 50 bp. The PCR reaction conditions were as follows: initial denaturation at 95 °C for 3 min, 8 cycles of denaturation at 98 °C for 15 s, annealing at 60 °C for 15 s, extension at 72 °C for 30 s, and then a final extension at 72 °C for 5 min. Finally, we performed 2 × 150 bp paired-end sequencing (PE150) on an Illumina Novaseq™ 6000 (LC-Bio Technology Co., Ltd., Hangzhou, China) following the vendor’s recommended protocol.

### 2.6. Bioinformatics Analysis

#### 2.6.1. Sequence and Filtering of Clean Reads

Using the Illumina paired-end RNA-seq approach, we sequenced the transcriptome, generating a total of million 2 × 150 bp paired-end reads. The raw reads obtained from the sequencer machines contained adapters or low-quality bases, which affected the following assembly and analysis. High-quality clean reads were obtained by Cutadapt filtering (https://cutadapt.readthedocs.io/en/stable/ (accessed on 17 March 2022), version: cutadapt-1.9). The parameters were as follows: (a) removing reads containing the adapters polyA and polyG; (b) removing reads containing more than 5% unknown nucleotides (N); (c) removing low-quality reads (more than 20% low-quality bases: Q-value ≤ 10); and (d) statistics of raw sequencing data, effective sequencing data, Q20, Q30, and GC content, and comprehensive evaluation.

#### 2.6.2. Analysis of RNA-Seq Data and Differentially Expressed Genes

We aligned reads of all samples to the reference genome using the HISAT2 (https://daehwankimlab.github.io/hisat2/ (accessed on 17 March 2022), version: hisat2-2.0.4) package. The mapped reads of all samples were assembled using StringTie software (default parameters) (https://ccb.jhu.edu/software/hisat2 (accessed on 17 March 2022), version: stringtie-1.3.4d). Then, a comprehensive transcript was reconstructed using gffcompare software (http://ccb.jhu.edu/software/stringtie/gffcompare.shtml (accessed on 17 March 2022), version: gffcompare-0.9.8). After the final transcriptome was generated, the expression level of the transcripts was evaluated by StringTie and ballgown, and the expression abundance of mRNA was calculated using the FPKM (fragment per kilobase of transcript per million mapped reads) value. The differential expression of genes between two groups of samples (y-Sham vs. y-PF; o-Sham vs. o-PF; y-Sham vs. o-Sham; and y-PF vs. o-PF) were analyzed using DESeq2 software [11,12]. The genes with a *q*-value (corrected *p*-value) ≤ 0.05 and absolute fold change (FC) ≥ 2 were considered differentially expressed genes. Differentially expressed genes were then subjected to enrichment analysis of their GO functions and KEGG pathways.

#### 2.6.3. GO and KEGG Enrichment Analysis

Differentially expressed genes were subjected to enrichment analysis of their Gene Ontology (GO) functions and Kyoto Encyclopedia of Genes and Genomes (KEGG) pathways. GO functional significant enrichment analysis was based on mapping all significantly differentially expressed genes to each term of the Gene Ontology database, calculating the number of genes per term, and then applying hypergeometric tests to identify GO entries that were significantly enriched in significantly differentially expressed genes compared with the entire genomic background [13,14]. KEGG is the main public database containing information on pathways, and pathway enrichment analysis was performed using “KEGG Pathway” as the unit and applying hypergeometric tests to identify pathways that were significantly enriched in significantly differentially expressed genes compared with the whole genomic background [15].

### 2.7. Statistical Analysis

All the experimental data are expressed as the mean ± SD (standard deviation) from at least three independent experiments. Data between the two groups were compared using a two-tailed Student’s *t*-test. *p*-values < 0.05 were considered to be statistically significant.

## 3. Results

### 3.1. Pulmonary Fibrosis in Old Rats Is More Serious than That in Young Rats

**Figure 1:**
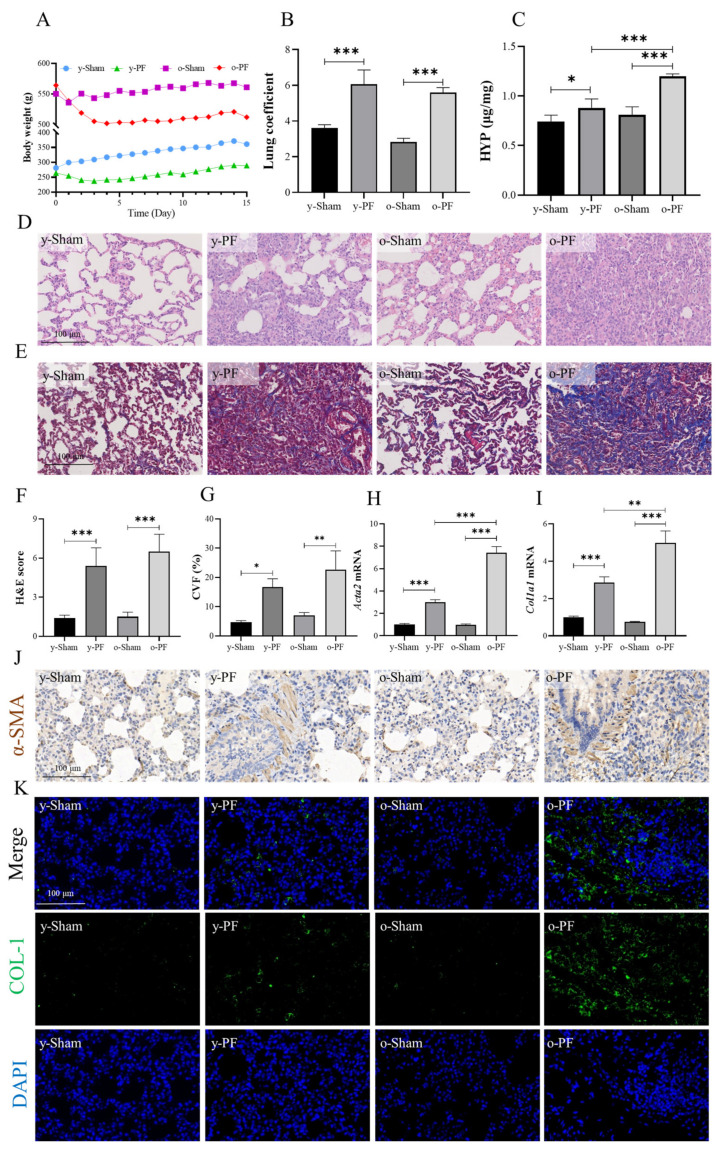
Pathological index of pulmonary fibrosis in young and old rats. The rats were treated with bleomycin (1 mg/kg) at day 0 and sacrificed on day 14. B (**A**) Body weight was recorded every day, and (**B**) lung coefficient and (**C**) HYP level in lung tissuewas determined on day 14. Representative images (200×) of lung tissue stained with (**D**) H&E or (**E**) Masson’s Trichrome were quantitatively assessed by (**F**) H&E scores or (**G**) CVF. The mRNA expressions of (**H**) *Acta2* (α-SMA) and (**I**) *Col1a1* (COL-1) were determined by q-PCR. Representative images (200×) of lung tissue (**J**) immunohistochemically stained with α-SMA or (**K**) immunohistofluorescently stained with COL-1. Data are expressed as mean ± SD (n = 3). * *p* < 0.05, ** *p* < 0.01, and *** *p* < 0.001.

Rats were treated with bleomycin at day 0 and sacrificed on day 14 for analysis of the pathological index. The average body weights of rats in the y-Sham and o-Sham groups increased steadily or remained stable. The average body weight of the y-PF group decreased in the first 3 days after bleomycin treatment and increased from day 4. However, the average body weight of the o-PF group decreased steadily until 14 days (Figure 1A). Lung coefficients are calculated by (lung wight × 1000)/bodyweight, which reflects the degree of pulmonary edema and inflammation. The lung coefficients of rats in the y-PF group were significantly increased compared with those of the y-Sham group (*p* < 0.001), and the lung coefficients of the o-PF group were also significantly increased compared with those of the o-Sham group (*p* < 0.001) (Figure 1B). Compared with the y-Sham group, the HYP level, H&E scores, and CVF of the y-PF group were significantly increased (*p* < 0.05 and *p* < 0.001). Similarly, the indexes of the o-PF group were significantly increased compared with those in the o-Sham group (*p* < 0.01 and *p* < 0.001). In addition, compared with the y-PF group, the HYP level of the o-PF group also increased significantly (*p* < 0.001) (Figure 1C–G), which indicated that the degree of pulmonary fibrosis in old rats is more serious than that in young rats under the same conditions. Collagen I (COL-1) and α-SMA are biomarkers of many fibrotic diseases. In the present research, the mRNA and protein levels of lung tissues in rats were determined by real-time q-PCR and immunohistofluorescence analysis. The mRNA expression of Collagen I (Col1a1) and α-SMA (Acta2) in the PF groups (y-PF and o-PF) was significantly upregulated compared with the sham groups (y-Sham and o-Sham) (*p* < 0.001), and compared with the y-PF group, the mRNA expression of Col1a1 and Acta2 was significantly upregulated in the o-PF group (*p* < 0.01 and *p* < 0.001) (Figure 1H,I). In addition to the mRNA level, as shown in Figure 1J,K, the expression of α-SMA and COL-1 in the y-PF and o-PF groups was more than that in the y-Sham and o-Sham groups, and the area of biomarker expression in the o-PF group was more than that in the y-PF group. The results shown in Figure 1 demonstrate that the symptoms of pulmonary fibrosis are more severe in old rats than in young rats, indicating that the elderly are more susceptible to pulmonary fibrosis.

### 3.2. Analysis of Differentially Expressed Genes between PF Groups and Sham Groups, or Young Groups and Old Groups

**Figure 2:**
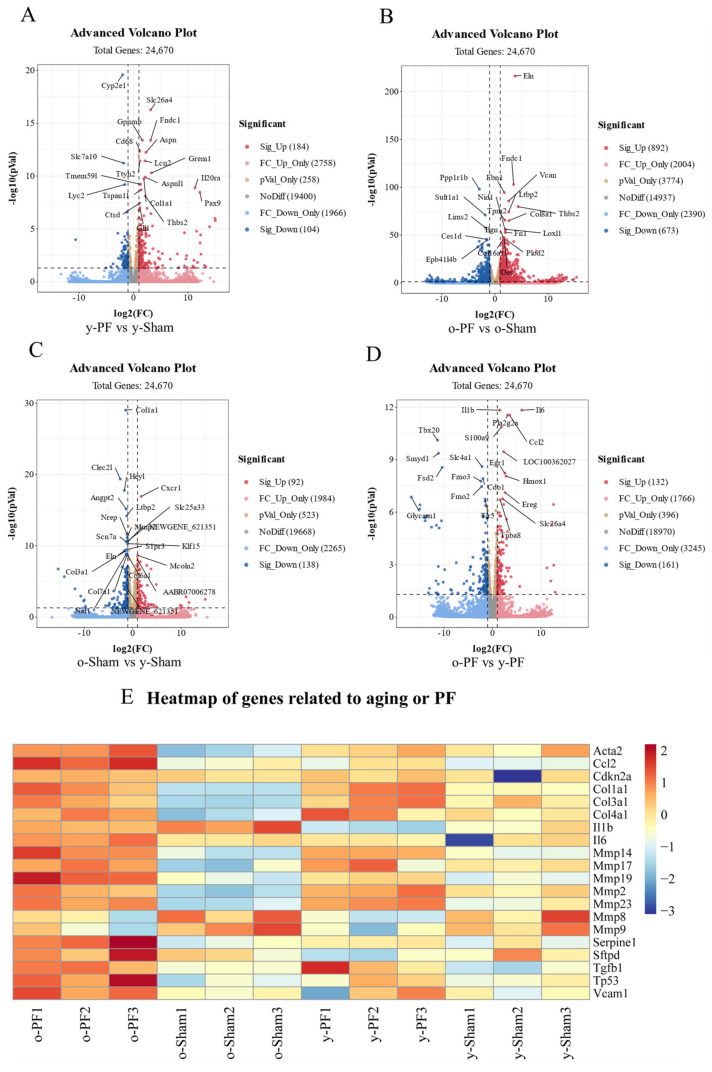
Analysis of differentially expressed genes between different groups. (**A**) Volcano plot for differentially expressed genes of y-PF vs. y-Sham. (**B**) Volcano plot for differentially expressed genes of o-PF vs. o-Sham. (**C**) Volcano plot for differentially expressed genes of o-Sham vs. y-Sham. (**D**) Volcano plot for differentially expressed genes of o-PF vs. y-PF. (**E**) Heatmap of genes related to PF and aging.

In order to investigate the differences in the mechanism of pulmonary fibrosis between young and old rats, the lung tissues of both groups were analyzed by transcriptome sequencing. The differential expression of mRNA derived from the two groups was then displayed as volcano plots, as shown in Figure 2A–D. Compared with the y-Sham group, 184 mRNAs were significantly upregulated (FC ≥ 2 and *q* < 0.05), and 104 mRNAs were significantly downregulated (FC ≤ 0.05 and *q* < 0.05) in the y-PF group (Figure 2A); compared with the o-Sham group, 892 mRNAs were significantly upregulated (FC ≥ 2 and *q* < 0.05) and 673 mRNAs were significantly downregulated (FC ≤ 0.05 and *q* < 0.05) in the o-PF group (Figure 2B). Then, in terms of age, compared with the y-Sham group, 92 mRNAs were significantly upregulated (FC ≥ 2 and *q* < 0.05), and 138 mRNAs were significantly downregulated (FC ≤ 0.05 and *q* < 0.05) in the o-Sham group (Figure 2C); compared with the y-PF group, 132 mRNAs were significantly upregulated (FC ≥ 2 and *q* < 0.05) and 161 mRNAs were significantly downregulated (FC ≤ 0.05 and *q* < 0.05) in the o-PF group (Figure 2D). A heatmap of the differential expression of mRNAs closely related to pulmonary fibrosis and senescence-associated secretory phenotype (SASP) is shown in Figure 2E. 

### 3.3. Enrichment of GO and KEGG Pathways of Differentially Expressed Genes between Sham and PF Groups

**Figure 3:**
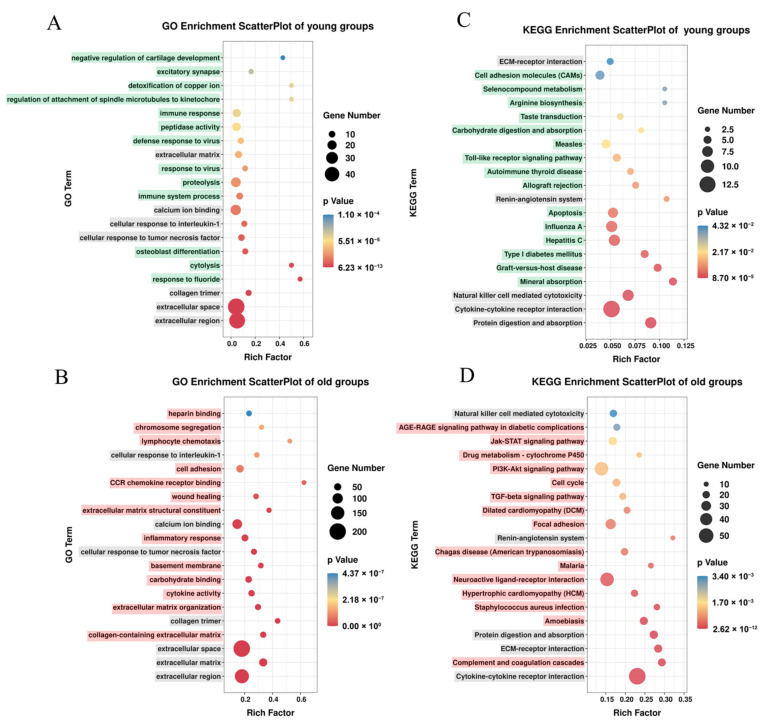
GO and KEGG enrichment analysis between different groups. (**A**) GO enrichment scatterplot of y-PF vs. y-Sham. (**B**) GO enrichment scatterplot of o-PF vs. o-Sham. (**C**) KEGG enrichment scatterplot of y-PF vs. y-Sham. (**D**) KEGG enrichment scatterplot of o-PF vs. o-Sham. The gray-highlighted terms are enriched both in young and old groups, the green-highlighted terms are only enriched in young groups, the red-highlighted terms are only enriched in old groups.

Bioinformatics analysis was performed to interpret the biological functions and related signaling pathways of differentially expressed mRNAs. The GO biological process, cellular component, and molecular function analysis showed that the differentially expressed mRNAs of y-Sham vs. y-PF groups were closely related to the extracellular region, extracellular space, collagen trimer, etc. (Figure 3A). The differentially expressed mRNAs of the o-Sham vs. o-PF groups were closely related to the extracellular region, extracellular matrix, extracellular space, etc. (Figure 3B). Among the GO enrichment terms, seven terms were overlapped (in gray) between the young groups (y-Sham vs. y-PF) and the old groups (o-Sham vs. o-PF), and the left terms (in green) were different between young groups and old groups. In the young groups, terms related to the immune response, such as immune system process, response, and response to the virus, were enriched for the differentially expressed mRNAs between the y-Sham and y-PF groups. Meanwhile, in the old groups, cytokine activity, inflammatory response, CCR chemokine receptor binding, chromosome segregation, and lymphocyte chemotaxis were enriched for the differentially expressed mRNAs between the o-Sham and o-PF groups. 

The KEGG pathway enrichment suggested that differentially expressed mRNAs might be involved in the enriched signal pathways, which play roles in pulmonary fibrosis. In the young groups, the enriched pathways mainly contained cytokine−cytokine receptor interactions, protein digestion, and absorption, natural-killer-cell-mediated cytotoxicity pathways, etc. (Figure 3C). In the old groups, the enriched pathways contained cytokine−cytokine receptor interactions, ECM−receptor interactions, cell cycle, the PI3K−Akt, AGE-RAGE, and TGF−beta signaling pathways, etc. (Figure 3D). Comparing the differences in signal pathways enriched from the differentially expressed mRNAs of the young groups and the old groups, we found that there were five pathways (in gray) involved in pulmonary fibrosis both in young and old rats and the remaining enriched signal pathways (in red) were quite different between young and old rats. In the young groups, it was suggested that mineral absorption, apoptosis, autoimmune thyroid disease, and Toll-like receptor signaling pathways were involved in pulmonary fibrosis. In the old groups, it was suggested that complement and coagulation cascades, neuroactive ligand−receptor interactions, and the PI3K−Akt, AGE-RAGE, cell cycle, and drug metabolism−cytochrome P450 pathways were involved in pulmonary fibrosis. By comparing the differences in GO and KEGG enrichment between young and old rats, it is possible to predict the differences in the mechanism of pulmonary fibrosis between young and old individuals. 

### 3.4. Screening and Analysis of Genes Associated with Both Pulmonary Fibrosis and Aging

**Figure 4:**
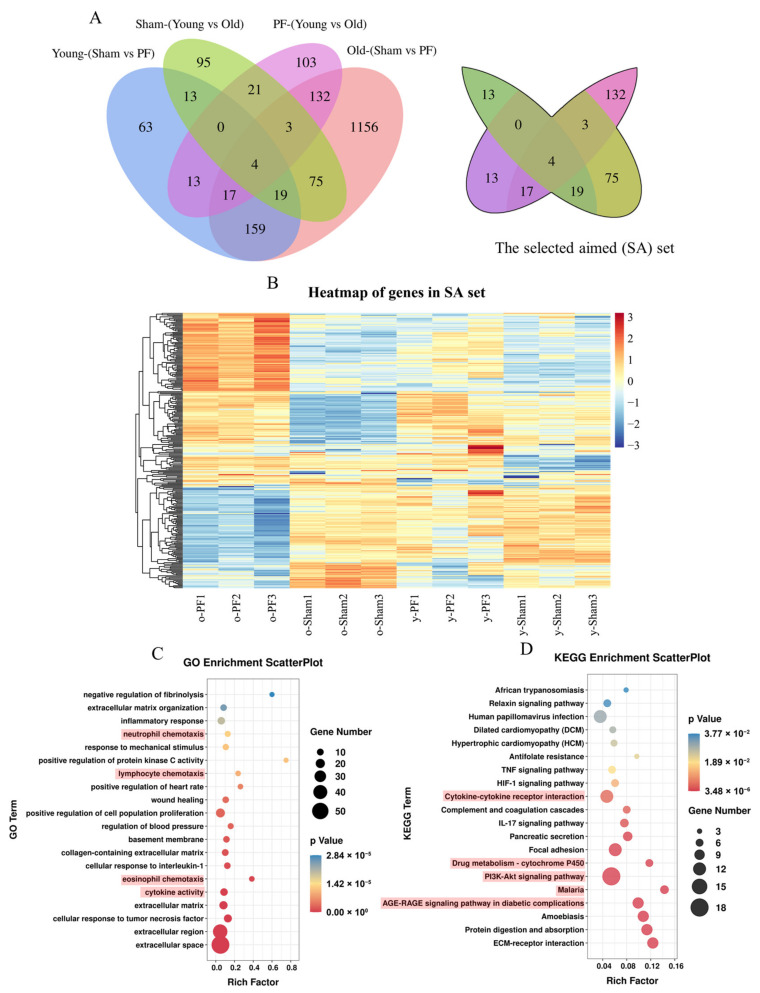
Screening and analysis of genes associated with both pulmonary fibrosis and aging. (**A**) Wayne diagram of four comparison sets and the selected set (SA). (**B**) Heatmap of the genes in SA set. (**C**) Go and (**D**) KEGG enrichment scatterplot of the genes in SA set.

In order to further explore the genes closely related to age and pulmonary fibrosis, a Wayne diagram was drawn to obtain the intersection and union of the four sets, Young (Sham vs. PF), Old (Sham vs. PF), Sham (Young vs. Old), and PF (Young vs. Old) (Figure 4A). As shown in Figure 4A, the differentially expressed mRNA sets of Young (Sham vs. PF) and Old (Sham vs. PF) were gathered; meanwhile, the mRNA sets of Sham (Young vs. Old) and PF (Young vs. Old) were also gathered. Then, the union of the sets of Young (Sham vs. PF) and Old (Sham vs. PF) was taken to intersect with the union of the sets of Sham (Young vs. Old) and PF (Young vs. Old), and the new intersection was selected, as shown in Figure 4A. The differentially expressed mRNAs of the selected aimed (SA) set were considered to be closely related to both pulmonary fibrosis and aging; a heatmap is shown in Figure 4B. The GO and KEGG enrichment of the SA set was performed to explore the relationship between these genes and their biological functions and signal pathways (Figure 4C,D). The GO enrichment indicated that the differentially expressed mRNAs of the SA set were closely related to extracellular space, extracellular region, the positive regulation of protein kinase C activity, and the negative regulation of fibrinolysis, etc. (Figure 4C). The KEGG enrichment indicated that the differentially expressed mRNAs of the SA set were closely related to ECM−receptor interactions, the PI3K−Akt, AFE-RAGE signaling pathway, cytokine−cytokine receptor, and drug metabolism−cytochrome P450 interactions, etc. (Figure 4D). 

### 3.5. Validation of Related Indicators: Biomarkers of Senescence and Senescence-Associated Secretory Phenotype (SASP) in Aged and Young Rats 

**Figure 5:**
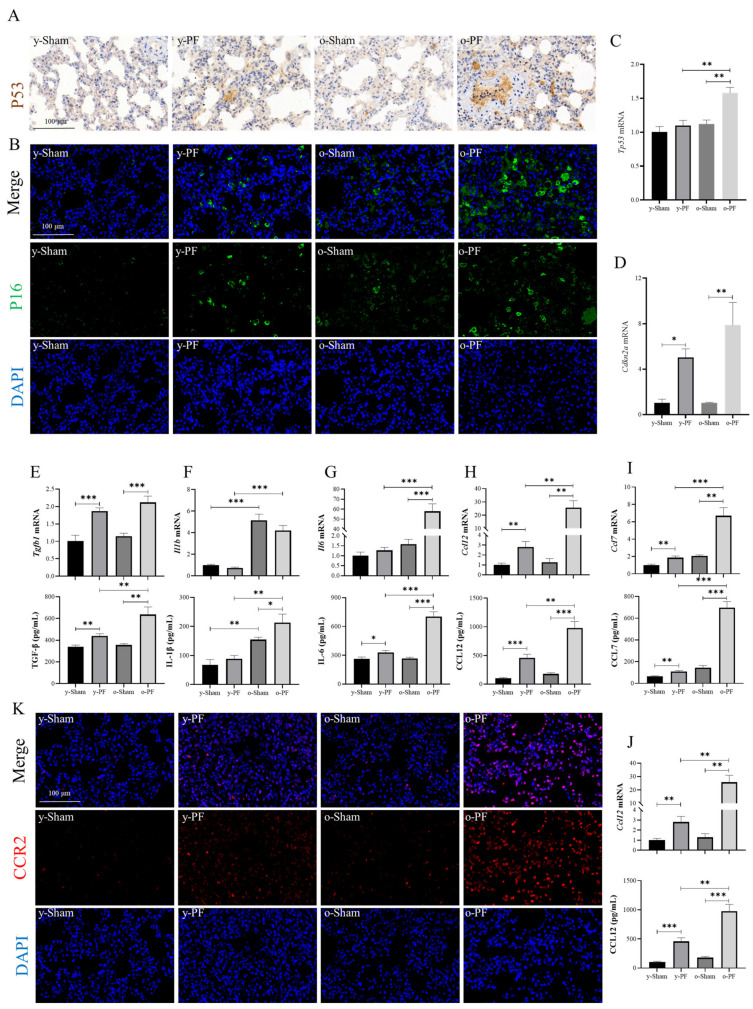
The mRNA and protein expressions of the main indexes related to PF and aging. Representative images (200×) of lung tissue (**A**) immunohistochemically stained with p53 antibody or (**B**) immunohistofluorescently stained with p16 antibody. The mRNA expressions of (**C**) *Tp53* (p53) and (**D**) *Cdkn2a* (p16) were determined by q-PCR. The mRNA and protein expressions of (**E**) TGF-β, (**F**) IL-1β, (**G**) IL-6, (**H**) CCL2, (**I**) CCL7, and (**J**) CCL12 were determined by q-PCR and ELISA. (**K**) Representative images (200×) of lung tissue immunohistofluorescently stained with CCR2 antibody. Data are expressed as mean ± SD (n = 3). * *p* < 0.05, ** *p* < 0.01, and *** *p* < 0.001.

From the results of previous GO and KEGG analyses, shown in Figure 3 and Figure 4, it can be seen that the cytokine−cytokine receptor interaction played an important role in pulmonary fibrosis and aging. Then, the mRNA and protein levels of biomarkers of senescence and several major cytokines involved were measured. The p16 and p53 are usually regarded as biomarkers of cell senescence and were highly expressed in the alveolar type II epithelial cells (AEC2) of patients with IPF [6,16,17]. As shown in Figure 5A,B, p16, and p53 were highly expressed in the y-PF and o-PF groups, and the expression level in the o-PF group was higher than that in the y-PF group. In addition, the mRNA of p53 (*Tp53*) expression in the o-PF group was significantly upregulated compared with the o-Sham group (*p* < 0.01) or the y-PF group (*p* < 0.01) (Figure 5C). Additionally, the mRNA of p16 (C*dkn2a*) expression in PF groups (y-PF and o-PF) was significantly upregulated compared with the sham groups (y-Sham and o-Sham) (*p* < 0.05 and *p* < 0.01) (Figure 5D). In the development of pulmonary fibrosis, senescent cells usually affect other cells via the secretion of senescence-associated secretory phenotype (SASP), which consists of multiple inflammatory proteins [18,19]. The mRNA and protein levels of several major cytokines in SASP, including TGF-β, IL-1β, IL-6, CCL2, CCL7, and CCL12, were determined by q-PCR and ELISA (Figure 5E–J). The mRNA expression of TGF-β was significantly upregulated in the y-PF group compared with the y-Sham group (*p* < 0.001), as well as the o-PF group vs. the o-Sham group (*p* < 0.001). The mRNA of the IL-1β group was significantly upregulated in the o-Sham group (*p* < 0.01 and *p* < 0.001), as well as the o-PF group vs. the y-PF group (*p* < 0.001), indicating the high expression of IL-1β mRNA in old rats, but the protein level of IL-1β was upregulated in the o-PF group compared with the o-Sham group (*p* < 0.05) or y-PF group (*p* < 0.01). The protein levels of TGF-β and mRNA and the protein levels of IL-6, CCL2, CCL7, and CCL12 were significantly upregulated in the y-PF group compared with the y-Sham group (*p* < 0.01 and *p* < 0.001). In addition, the protein levels of TGF-β and the mRNA and protein levels of IL-6, CCL2, CCL7, and CCL12 were significantly upregulated in the o-PF group compared with the o-Sham group (*p* < 0.01 and *p* < 0.001) or o-PF group (*p* < 0.01 and *p* < 0.001). However, the upregulated fold changes in these cytokines between the y-PF and y-Sham groups were much lower than those between the o-PF and o-Sham groups. CCR2 is the receptor of CCL2, CCL7, and CCL12, which plays an important role in the progression of pulmonary fibrosis [20,21]. The CCR2 expression in lung tissues was investigated by immunohistofluorescence (Figure 5K). Consistent with its ligands, CCR2 was highly expressed in the PF groups and was little expressed in the sham groups, and the expression in the o-PF group was the highest. 

## 4. Discussion

Aging has always been considered an important factor in fibrosis diseases, but the specific effects of aging on fibrosis have not been fully clarified [22]. In the present study, the basic indexes of animal experiments showed that old rats are more sensitive to lung injury than young rats, which suggests that the elderly are more likely to suffer from pulmonary fibrosis. Consistent with this suggestion, the mRNAs displayed in Figure 2E, which were closely related to pulmonary fibrosis and senescence, were more highly expressed in the o-PF group. According to the analysis of the transcriptome and bioinformatics, we found that there was a great difference in gene transcripts between old rats and young rats during the occurrence of pulmonary fibrosis, which warrants further analysis. Between the two young groups, 288 genes exhibited a significant difference, compared with 1565 genes between the two old groups, indicating that in elderly individuals, when fibrosis occurs, the changes in the body are more complex than those in the young. 

The abnormal proliferation of the extracellular matrix (ECM) is the direct cause of fibrosis [2]. Thus, the GO enrichment shows that the terms extracellular matrix, extracellular space, etc., are significantly enriched in both young and old groups (Figure 3A,B). However, for the same term, the numbers of differentially expressed genes attributed to ECM in young rats differ from those in old rats, there being 12 ECM-related differentially expressed genes in young groups and 65 ECM-related differentially expressed genes in old groups. Most of these mRNAs were regulated in PF groups (Tables S3 and S4). the upregulation of more ECM-related genes means that there are more opportunities for ECM proliferation in elderly individuals, thus causing pulmonary fibrosis. In terms of the GO enrichment of old rats, chemokine- or cytokine-related terms accounted for a large proportion, which was not enriched in young rats. Alveolar epithelial cell injuries are thought to initiate the expansion of myofibroblasts and the excessive deposition of ECM, and cytokines and chemokines play important roles as pathogenetic mediators of pulmonary fibrosis [23,24]. Additionally, the term “chromosome segregation” was enriched in old rats, and the genes were almost upregulated in the o-PF group, such as centromere protein (CENPs), NIMA-related kinase (NEKs) and *Pttg1,* etc., which indicated the genes related to chromosome segregation might be involved in the progression of PF (Table S4). The abnormal regulation of genes related to chromosome segregation can lead to chromosome instability and chromosome segregation errors, which results in cell cycle arrest and senescence [25,26,27,28]. In old rats, the mRNAs of chemokine- and cytokine-related terms are significantly upregulated in the o-PF group, indicating the imbalance of immune regulation or chromosome segregation error in old rats with pulmonary fibrosis, thus aggravating the process of pulmonary fibrosis. 

The KEGG pathway enrichment shows that the pathway with the largest number of enriched genes is the cytokine−cytokine receptor interaction pathway in both young and old rats. However, in old rats, 59 cytokines or cytokine receptors are changed in the o-PF group vs. the o-Sham group, and the numbers of changed cytokines or cytokine receptors in the y-PF vs. the y-Sham group are less than in the o-PF group vs. the o-Sham group, which is similar to the results of the GO enrichment (Tables S5 and S6). These results once again show the importance of cytokines in pulmonary fibrosis in old rats. The most significant pathway enriched by KEGG is the ECM−receptor interaction containing the genes Itga11, Itgb8, Thbs1, Thbs2, etc. (Table S9). There is extensive crosstalk between integrins and TGF-β signaling, as evidenced by the ability of TGF-β to affect integrin-mediated cell adhesion and migration and the ability of integrins to directly activate TGF-β [29,30]. As a performer in pulmonary fibrosis, TGF-β is activated by enzymes such as thrombospondin-1 for the following pro-fibrotic effects [31]. The mRNA expression of Itga11, Itgb8, Thbs1, and Thbs2 was significantly upregulated in the o-PF group, indicating the stronger activity of TGF-β in old rats when pulmonary fibrosis occurs. Interestingly, thrombospondin-1 deficiency cannot prevent pulmonary fibrosis in the bleomycin-induced model, which suggests that thrombospondin-1 is not essential for TGF-β activation [32]. In addition, PI3K-AKT and Jak−STAT are the main signaling pathways involved in the pathogenesis of pulmonary fibrosis in old rats. The PI3K-AKT signaling pathway is involved in the regulation of vital biological processes such as cell growth, proliferation, movement, metabolism, and survival [33]. In the process of pulmonary fibrosis, PI3K-AKT interacts with TGF-β to promote the overexpression of a-SMA [34]. In addition, the activation of the PI3K-AKT pathway can regulate downstream targets such as HIF-1α, mTOR, and the FOX family to participate in the pathogenesis of fibrosis [34]. The Jak−STAT pathway is activated by several cytokines, such as IL-6, IL-4, IL-13, and TGF-β, which are implicated in the pathogenicity of pulmonary fibrosis [35]. STAT3 regulates IL-6- and TGF-β-mediated myofibroblast differentiation and the epithelial-to-mesenchymal transition (EMT) in pulmonary fibrosis [36,37]. In addition, IL-6 and STAT3 are related to the induction of senescence, and STAT3 is a key factor of oxidant-induced senescence in lung fibroblasts [38,39]. Notably, the AGE-RAGE signaling pathway was also enriched in KEGG analysis of aged rats (Figure 3D). In our results, the genes belonging to the “AGE-RAGE pathway” enriched in KEGG analysis of old rats can be roughly divided into two categories: ECM (*Col1a1*, *Col3a1*, *Col4a*, and *Fn1,* etc.) and SASP (*Il6*, *Ccl2*, *Ccl12*, and *Serpine1,* etc.), which were up-regulated in the o-PF group compared to o-Sham group (Table S6). The advanced glycation end-products (AGEs) are the consequence of non-enzymatic reactions between lipids and proteins with several oxidants in the aging process [40]. The Receptor for advanced glycation end products (RAGE) is highly expressed in alveolar epithelial cells, which play a homeostatic role in the lung [41,42]. AGE-modified collagen leads to an increase in matrix stiffness, which makes it resistant to hydrolytic turnover, and ultimately results in an accumulation of extracellular matrix (ECM) proteins [43]. And the interactions of AGEs and RAGE initiate pro-inflammatory signaling, oxidative stress, and senescence, which result in the release of pro-inflammatory and pro-fibrosis cytokines [44,45]. In addition, RAGE expression decreases with the development of pulmonary fibrosis, which is confirmed in the animal model and IPF patients [42,44]. However, RAGE is essential for efficient double-strand break (DSB)-repair via the ataxia-telangiectasia-mutated ATM signaling cascade [46,47]. The downregulated RAGE indicates the inadequate ability for DSBs repair, which aggravates the DNA damage response in alveolar epithelial cells. Additionally, “cell cycle” pathway was enriched in old rats, all of which genes were up-regulated in o-PF group (Table S6). The biomarkers of senescence, p53 and p16, which are highly expressed in o-PF group (Figure 5A–D), are characteristic indexes of cell cycle arrest, and the upregulation of cyclin-dependent kinases (CDKs) (Table S6) also promotes cell cycle arrest. In addition, the cell cycle is closely related to the chromosome segregation mentioned in the previous GO enrichment, which is involved in the release of SASP and result in the development of fibrosis [26,48]. The results of KEGG pathway analysis indicate that the changed cytokines mainly regulate pulmonary fibrosis in old rats related to the PI3K/Akt, Jak/STAT, AGE-RAGE and cell cycle signaling pathways.

According to the Wayne diagram of different gene sets between different comparison groups, we obtained the SA set, the genes which may be related to both pulmonary fibrosis and aging (Figure 4B and Table S7). From the cluster heatmap of the SA set, it can intuitively be seen that the mRNA expression of these genes in the o-PF group is much higher or much lower than that in the remaining three groups, indicating that aging affects the mRNA expression of these genes during pulmonary fibrosis. Chemokine- and cytokine-related terms are mainly enriched in the GO enrichment of the SA set. Then, we determined several cytokines or chemokines in the lung tissues of rats. The results of ELISA and the q-PCR indicated that the protein and mRNA expression of the cytokines is similar to that in the heatmap (Figure 4B and Figure 5E–J). Among the pathways enriched by the KEGG enrichment analysis of the SA set, the PI3K/AKT, HIF-1, AGE-RAGE, and drug metabolism−cytochrome P450 pathways are reported to be associated with aging and pulmonary fibrosis. Compared with the PI3K/AKT and HIF-1 pathways, which are widely known to be associated with aging and fibrotic diseases, the drug metabolism−cytochrome P450 pathway has not been widely reported [34,49,50]. The enriched genes of the drug metabolism−cytochrome P450 pathway of the SA set contain several members of the flavin-containing monooxygenase (FMO) family, Fmo2, Fmo3, and Fmo6, which are downregulated in the o-PF group compared with other three groups (Tables S7 and S9). Flavin-containing monooxygenases (FMOs) are xenobiotic-metabolizing enzymes which have a role in promoting health and longevity [51]. The upregulation of FMOs prevents or reverses hepatic aging by promoting autophagy. On the contrary, the downregulation of FMOs is also recognized as a biomarker of aging [51,52]. In addition, FMO2 suppresses Smad2/3 signaling by binding to CYP2J3 and promoting the translocation of SMURF2 (SMAD-specific E3 ubiquitin ligase 2) interacting with CYP2J3, which plays an anti-fibrotic role [53]. The downregulated FMOs in old rats indicate a decline in their anti-fibrosis ability. The “AGE-RAGE pathway” was enriched in the SA set, indicating that the active AGE-RAGE cascade might be responsible for higher cytokines and higher ECM levels in older rats during pulmonary fibrosis. In addition, the Malaria pathway had the highest enrichment factor among all enriched pathways of the SA set (Figure 4D). However, the enriched genes of the Malaria pathway of the SA set contained a series of cytokines such as IL-6, CCL2, and CCL12, which is consistent with the results of GO enrichment (Table S9). What’s more, comparing the o-PF and y-PF groups, the information found by GO and KEGG enrichment was similar to that of the SA set (Figure S1), with mainly the chemokine- and cytokine-related terms and the PI3K/AKT, HIF-1, and drug metabolism−cytochrome P450 pathways being enriched. However, comparing the o-Sham and y-Sham groups, the terms related to the immune response and macrophages were found to be enriched by GO enrichment, as indicated in Figure S1. Related genes were significantly upregulated in the o-Sham group (Table S10), suggesting that a sensitive immune response occurred in old rats. The PPAR pathway was significantly enriched in the KEGG enrichment of sham groups (o-Sham vs. y-Sham, Figure S1), and the related genes were significantly downregulated (Table S11). The downregulation of PPAR indicates that the differentiation, expansion, and fate commitment of immune cells are negatively affected, which leads to immune dysfunction [54,55].

In GO and KEGG enrichment analysis, the highest enrichment frequency was found for cytokines. Thus, the main cytokines were validated on the mRNA and protein levels. The p53 and p16 in the PF groups were higher than in the Sham groups, indicating that senescence occurred during pulmonary fibrosis (Figure 5A–D). In our research, the SASP IL-6 and TGF-β levels in the o-PF group were higher than in the other three groups, suggesting that high levels of pro-fibrotic cytokines aggravate pulmonary fibrosis in old rats. It has been reported that CCL2 promotes the activation of fibroblasts via ERK1/2-mediated IL-6 and suppresses the apoptosis of fibroblasts via the IL-6/STAT3 pathway [56,57]. Clinical research has shown that CCR2+ (CCL2 receptor) macrophages and monocytes are extremely active in the lung and are located in the central area of fibrosis of IPF patients, indicating that CCL2/CCR2 plays an important role in the pathogenesis of fibrosis [56]. The CCR2 has three ligands, CCL2, CCL7, and CCL12, secreted by AEC2 or vascular endothelial cells, which are elevated in the injured lung, and promote fibrosis via the CCR2/mTOR pathway [21,58]. The elevated CCL2, CCL7, and CCR2 in the lung of the o-PF group suggest they are the important executors of pulmonary fibrosis in old rats, in addition to TGF-β. As the classic inflammatory cytokine, IL-1β plays an important role in pulmonary fibrosis. The over-expression of IL-1β induces the expression of TGF-β, and IL-1β/caspase-1 initiates fibrosis after being injured by bleomycin, which is related to the activation of NLRP3, AIM2, and NLRC4 due to mitochondrial oxidative stress [59,60]. The expression of IL-1β in old rats is higher than that in young rats, and the high level of IL-1β increases the likelihood of the occurrence of fibrosis.

In conclusion, comparing the transcripts of the four groups, we found differences between the young and old rats. During the progression of pulmonary fibrosis, old rats have a higher risk of pulmonary fibrosis due to the presence of highly sensitive cytokines and chemokines, which may be affected by inadequate immune regulatory capacity, epigenetic changes, and the loss of anti-fibrotic targets in old rats.

## 5. Conclusions

In this study, both young and old rats were modeled for pulmonary fibrosis. The results indicate that the old rats suffered from more severe pulmonary fibrosis than the young rats. Comparing the changes in the transcripts of young and old rats during pulmonary fibrosis, we found that the highly expressed chemokines, ECM, pro-fibrotic genes, or the low expression of genes with anti-fibrotic ability contributed to the occurrence of fibrosis in old rats.

## Data Availability

Not applicable.

## Supplementary Materials

The following supporting information can be downloaded at: https://www.mdpi.com/article/10.3390/life12121961/s1, Table S1: Primer sequences for qPCR assay; Table S2: Heatmap of Figure 3E; Table S3: y-PF vs. y-Sham GO enrichment gene of Figure 3A; Table S4: o-PF vs. o-Sham GO enrichment gene of Figure 3B; Table S5: y-PF vs. y-Sham KEGG enrichment gene of Figure 3C; Table S6: o-PF vs. o-Sham KEGG enrichment gene of Figure 3D; Table S7: Heatmap of Figure 5B; Table S8: SA set GO enrichment gene of Figure 5C; Table S9: SA set KEGG enrichment gene of Figure 5D; Table S10: o-Sham vs y-Sham GO enrichment Gene of Figure S1A.; Table S11: o-Sham vs y-Sham KEGG enrichment Gene of Figure S1B; Table S12: o-PF vs y-PF GO enrichment Gene of Figure S1C; Table S13: o-PF vs y-PF KEGG enrichment Gene of Figure S1D; Figure S1: GO and KEGG enrichment analysis between old and young groups.
