# Supplementary material for: Analysis of the Potential Relationship between Aging and Pulmonary Fibrosis Based on Transcriptome
Source: Life (Basel). 2022 Nov 23;12(12):1961. doi: 10.3390/life12121961 (PMC9788318; doi:10.3390/life12121961)
Supplement: Supplementary file 1 [file life-12-01961-s001.zip › Figure S1-GO and KEGG enrichment analysis between old and young groups.pdf]

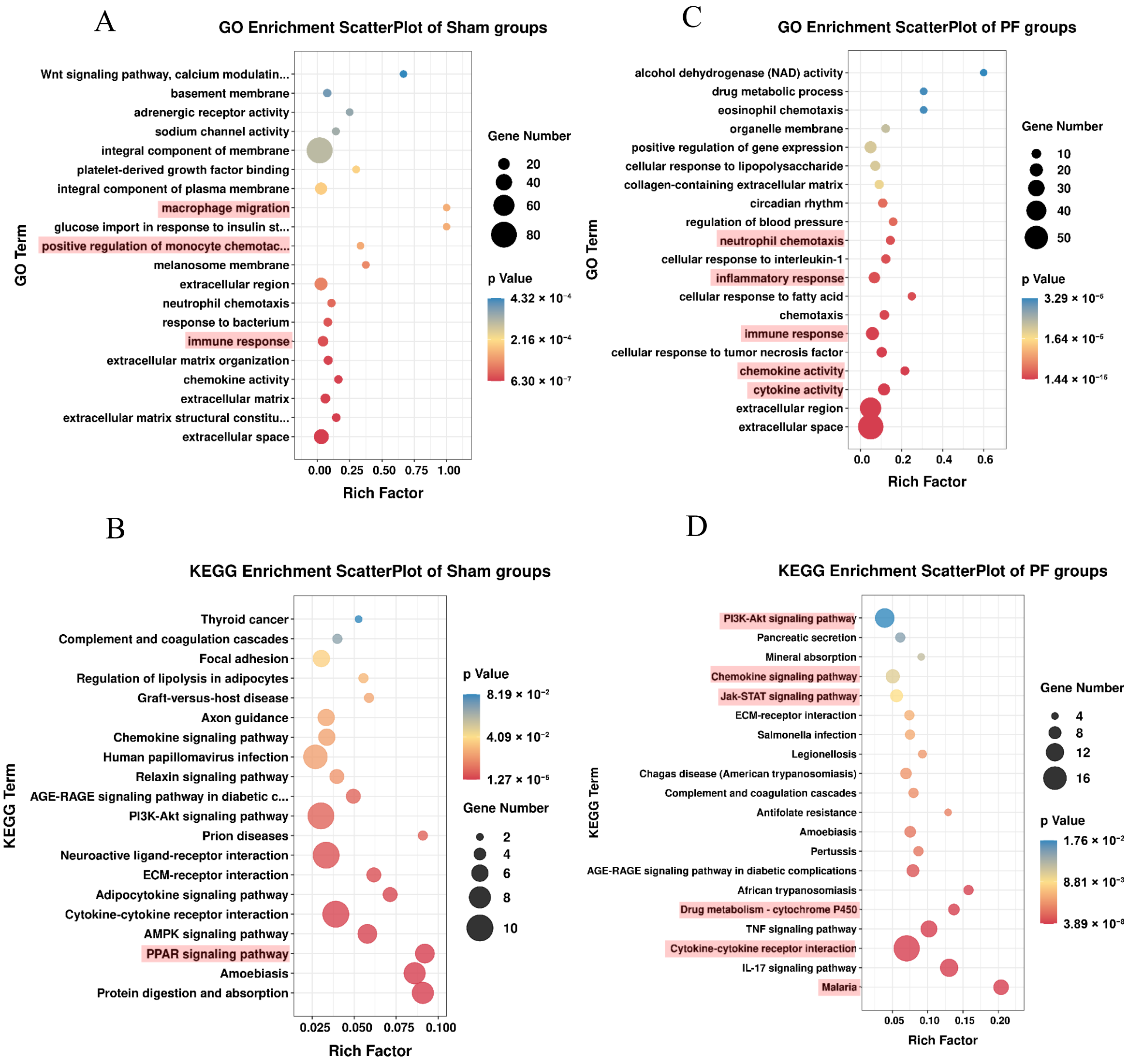

Figure S1. GO and KEGG enrichment analysis between old and young groups. (**A**) GO enrichment scatterplot of o-Sham vs. y-Sham. (**B**) KEGG enrichment scatterplot of o-Sham vs. y-Sham. (**C**) GO enrichment scatterplot of O-PF vs. y-PF. (**D**) KEGG enrichment scatterplot of o-PF vs. y-PF.
