# Supplementary material for: Analysis of the Potential Relationship between Aging and Pulmonary Fibrosis Based on Transcriptome
Source: Life (Basel). 2022 Nov 23;12(12):1961. doi: 10.3390/life12121961 (PMC9788318; doi:10.3390/life12121961)
Supplement: Supplementary file 1 [file life-12-01961-s001.zip › Table S1-Primer sequences for qPCR assay..pdf]

Table S1

Primer sequences for qPCR assay.

Gene name	Sequence		Length (bp)
Gapdh	Forward primer	5'- GTCGGTGTGAACGGATTTGG-3'	199
	Reverse primer	5'- TCCCGTTGATGACCAGCTTC-3'	
Acta2	Forward primer	5'-CATCCGACCTTGCTAACGGA-3'	366
	Reverse primer	5'-GTCCAGAGCGACATAGCACA-3'	
Col1a1	Forward primer	5'-GATGGACTCAACGGTCTCCC-3'	257
	Reverse primer	5'-CGGCCACCATCTTGAGACTT-3'	
Tp53	Forward primer	5'-CCAGGGAGTGCAAAGAGAGC-3'	320
	Reverse primer	5'- CTGGCCCTTCTTGGTCTTCG-3'	
Cdkn2a	Forward primer	5'-CGTGCGGTATTTGCGGTATCT-3'	178
	Reverse primer	5'-TCGCGTTGCCAGAAGTGAAG-3'	
Tgfb1	Forward primer	5'-ACGGAATACAGGGCTTTCGC-3'	298
	Reverse primer	5'-ATCCACTTCCAACCCAGGTC-3'	
Il1b	Forward primer	5'-TGACTTGGGCTGTCCAGATG-3'	221
	Reverse primer	5'-GGTCGTCATCATCCCACGAG-3'	
Il6	Forward primer	5'-GCACATGCCGTGACTTAAGACT-3'	71
	Reverse primer	5'-GGACACCCCTTCTGCGTTG-3'	
Il6	Forward primer	5'-CCCAACTTCCAATGCTCTCCT-3'	265
	Reverse primer	5'-GGATGGTCTTGGTCCTTAGCC-3'	
Ccl2	Forward primer	5'-CAGGTCTCTGTCACGCTTCT-3'	169
	Reverse primer	5'-TGCTGCTGGTGATTCTCTTGT-3'	
Ccl12	Forward primer	5'-ACCAGATTCAGTGTTCACCCC -3'	200
	Reverse primer	5'-CCAAGTGGTTCATGGAGTCCT -3'	
Ccl7	Forward primer	5'-TGCCTGAACAGAAACCAACC -3'	95
	Reverse primer	5'-TAGGACCGTAGTCCACCCATT -3'	
